# Percutaneous robot-assisted screw fixation for nondisplaced pelvic fractures: a good choice?

**DOI:** 10.1007/s00264-023-05794-x

**Published:** 2023-03-30

**Authors:** Zongdong Zhu, Bo Tan, Dan Wei, Xiaoming Tang, Jiabin Yuan, Jiang Hu, Feng Liao

**Affiliations:** Department of Orthopaedics, Sichuan Provincial People’s Hospital, University of Electronic Science and Technology of China, 32# W. Sec 2, 1st Ring Rd, Qingyang District, Chengdu, 610072 China

**Keywords:** Nondisplaced pelvic fracture, Robot-assisted surgery, Cannulated screw, Complication, Majeed score

## Abstract

**Purpose:**

To compare the merits and demerits of percutaneous robot-assisted screw fixation for nondisplaced pelvic fractures with other treatments via long-term follow-up.

**Methods:**

This was a retrospective analysis of nondisplaced pelvic fractures treated between January 2015 and December 2021. The number of fluoroscopy exposures, operative duration, intraoperative blood loss, surgical complications, screw placement accuracy and Majeed score were compared among the nonoperative group (24 cases), open reduction and internal fixation (ORIF) group (45 cases), free-hand empirical screw fixation (FH) group (10 cases) and robot-assisted screw fixation (RA) group (40 cases).

**Results:**

There was less intraoperative blood loss in the RA and FH groups than in the ORIF group. The number of fluoroscopy exposures in the RA group was lower than that in the FH group but much higher than that in the ORIF group. There were five cases of wound infection in the ORIF group and no surgical complications in the FH or RA group. The medical expenses were higher in the RA group than in the FH group, with no significant difference from the ORIF group. The Majeed score was lowest in the nonoperative group three months after injury (64.5±12.0) but lowest in the ORIF group one year after injury (88.6±4.1).

**Conclusion:**

Percutaneous RA for nondisplaced pelvic fractures is effective and minimally invasive and does not increase medical expenses compared with ORIF. Therefore, it is the best choice for patients with nondisplaced pelvic fractures.

## Introduction

Reconstruction of the stability of the pelvic ring is an important goal in the treatment of pelvic fractures. However, because the stability of nondisplaced pelvic fractures can only be judged by a stress test rather than by imaging, which may increase patient pain and cause further injury, it is difficult to decide whether such patients need early surgical treatment in clinical practice [1–3]. Nonoperative treatment takes a long time, and the effect is unsatisfactory [4, 5]. Open reduction and internal fixation (ORIF) of the pelvis using sacral bars or plating is traumatic and complicated, although it allows for earlier mobilization [6]. In recent years, percutaneous screw fixation has been shown to be effective, with lower risks of intraoperative blood loss, infection, and nerve and vascular injury [7, 8]. However, free-hand empirical screw placement under fluoroscopic monitoring is challenging due to the complex pelvic anatomy and requires skilful correlation of fluoroscopic images and bony landmarks [9–11].

With the development of and advances in medical imaging and computer technologies, navigation- and robot-assisted minimally invasive internal fixation methods have been applied to assist in orthopaedic surgery. Recently, with the combination of stereotactic and automatic manipulation methods, versatile state-of-the-art robot-based navigation systems for orthopaedic surgery have been developed, such as the “TianJi” robotic system. Given the advantages of being simple and minimally invasive, enabling precise positioning, and involving minimal radiation exposure, robot-assisted orthopaedic surgery is being accepted by an increasing number of doctors. A few studies have reported its advantages in pelvic fracture surgery [12, 13].

However, considering the medical costs, whether it is necessary to fix all nondisplaced pelvic fractures at an early stage with percutaneous screws remains controversial. Therefore, in this study, we intend to compare the merits and demerits of percutaneous robot-assisted screw fixation with other treatments through long-term follow-up to help doctors and patients make better choices.

## Materials and methods

### Inclusion and exclusion criteria

This study was approved by our institutional review board.

We retrospectively analysed all patients who were diagnosed with pelvic ring fractures between January 2015 and December 2021 at our institution. Anteroposterior, inlet and outlet X-ray views of the pelvis and computed tomography (CT) scans were routinely obtained from all patients. Patients were eligible for inclusion in this study if they (i) had pelvic ring injuries without fracture displacement (maximal displacement of anterior and posterior pelvic arch ≤4 mm measured on the three pelvic views [14]) and (ii) completed at least one year of follow-up. The exclusion criteria were as follows: (i) inability to cooperate with rehabilitation exercise or follow-up due to mental illness or other serious systemic diseases; (ii) incomplete data; or (iii) refusal to participate in this study.

The decision regarding the management of pelvic fractures is based on every patient's own wishes and physical condition, the surgical skill of the doctor, and the availability of equipment when the patient is admitted.

### Nonoperation

Patients who received nonoperative treatment underwent rehabilitation at home or in the hospital according to Association for the Study of Internal Fixation (AO) guidelines [15]. Briefly, the patient’s pelvis was fixed with a pelvic girdle, and the patient rested in bed while cranial or ovoid displacement of the fracture was reduced with skeletal traction. Oral medication was used to relieve pain, and early functional exercise was performed, as instructed by the physiotherapist, to prevent complications such as bedsores, deep venous thrombosis of the lower limbs, and falling pneumonia. X-ray or CT examinations were performed every month to evaluate fracture healing. Depending on the level of pain and fracture healing, mobilization was initiated with a walker and advanced to mobilization with crutches or a cane. The weight-bearing over the affected limb gradually increased until the fracture healed completely.

### Surgical procedure for ORIF

**Fig. 1:**
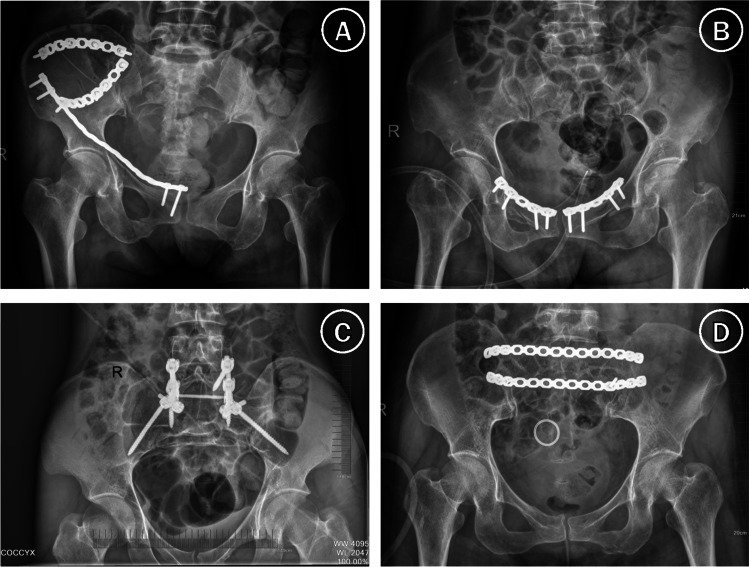
Postoperative pelvic X-ray images of nondisplaced pelvic fracture treated by ORIF. (**A**) Fixation of sacroiliac joint, ilium and superior pubic branch through anterior approach; first and third window of ilioinguinal approach. (**B**) Fixation of bilateral superior pubic branches via Stoppa approach. (**C**) Spino-pelvic fixation via posterior approach. (**D**) Ilioiliac plate fixation via posterior approach

According to AO guidelines, the posterior arch was fixed with two dynamic compression plates across the sacroiliac joint through an anterior approach [16] or ilioiliac plate/spino-pelvic fixation via a posterior approach [17, 18]. The anterior arch was repaired by a pubic ramus plate via a modified Stoppa approach [19] (Fig. 1).

### Surgical procedure for free-hand empirical screw fixation (FH)

Iliosacral screws and/or anterograde/retrograde pubic ramus screws were placed at the S1 and S2 levels according to AO guidelines [20, 21]. Briefly, the surgeon used a C-arm fluoroscope in conventional 2D mode. The guiding needles were adjusted according to the insertion location and angle and were gradually advanced under repeated optimal image intensification on two planes until the optimal anatomical location was reached. After manually measuring the length, a cannulated drill bit was used to make the appropriate canal. The iliosacral cannulated and anterograde/retrograde pubic ramus screws were then inserted along the guiding needles, and the skin and subcutaneous tissues were sutured.

### Surgical procedure for robot-assisted screw fixation (RA)

**Fig. 2:**
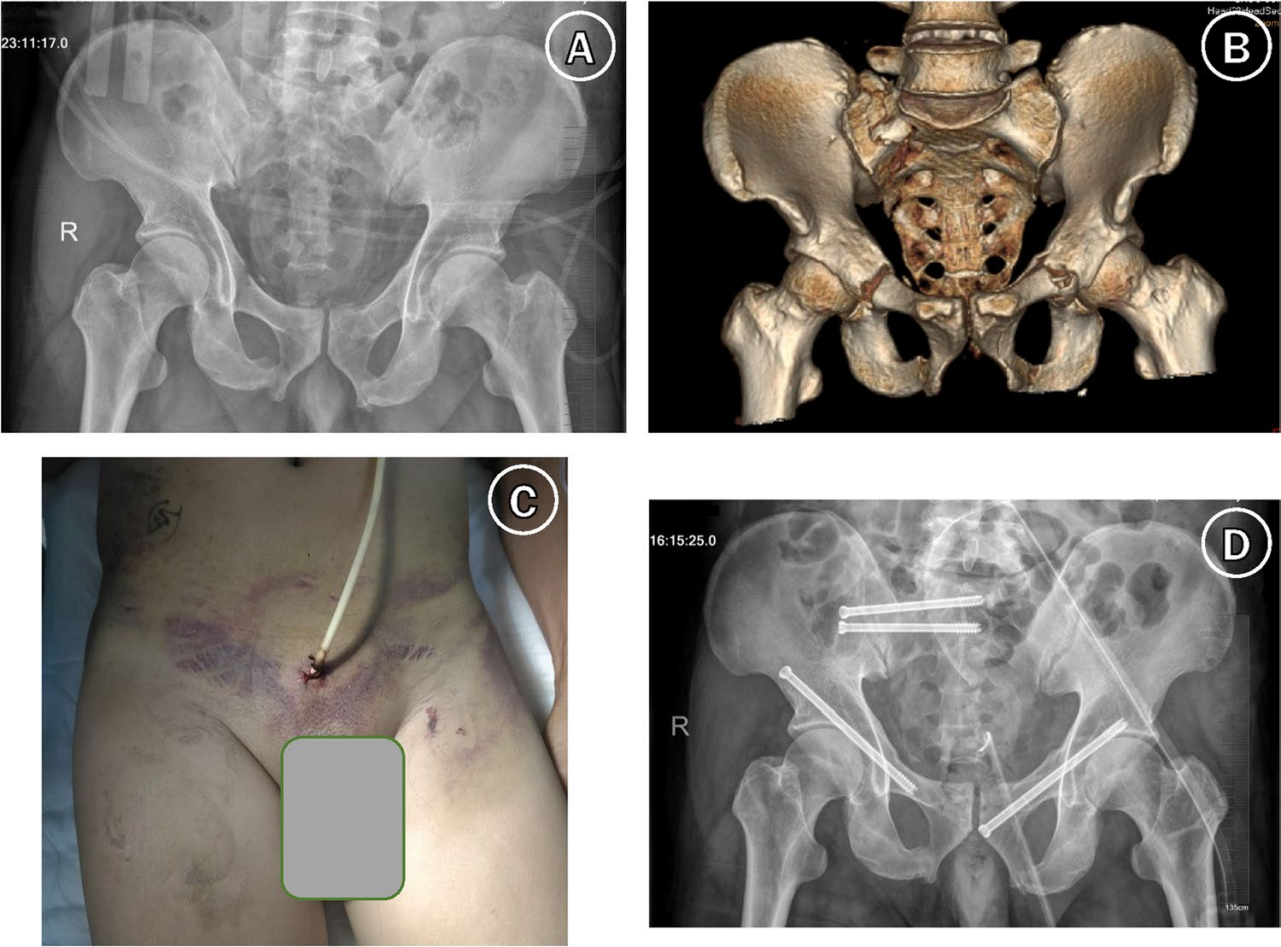
A 47-year-old man had an open nondisplaced pelvic fracture treated with RA. (**A**) Preoperative anteroposterior pelvic X-ray image. (**B**) Preoperative three-dimensional CT image. (**C**) The case was complicated with urethral injury, lung contusion, and fractures of the left tibia and fibula and right ankle. Emergency cystostomy was performed. (**D**) The patient was treated with percutaneous S1- and S2-level iliosacral screw and anterograde/retrograde pubic ramus screws fixation with the TianJi Robot system. He could sit up or turn over in bed after the operation, and urethral repair was successfully performed later

All operations were performed by the TianJi Robot (TiRobot) system (TINAVI Medical Technologies, Beijing, China). This system is composed of a mechanical arm, an optical tracking system, and a workstation for operative planning and control. All surgeries were performed by the same surgical team, which is very familiar with the TianJi Robot system. The steps for percutaneous S1- and S2-level iliosacral screw and/or anterograde/retrograde pubic ramus screw placement with the TianJi Robot system were performed as previously reported [12] (Fig. 2).

### Postoperative treatment

The postoperative regimens were similar in all groups. Prophylactic anti-infection treatment was administered for 24 hours after the surgery. Meanwhile, treatment for the prevention of deep vein thrombosis was maintained for five weeks. On the first day after the surgery, inlet, outlet, and anteroposterior pelvic radiographs, as well as CT scans, were obtained. If the internal fixation was successful, the patients were allowed to sit up and perform rehabilitation exercises with postoperative analgesia. Weight bearing on the uninjured side was not limited, while weight bearing on the injured side was determined according to the stability of internal fixation. For patients treated by percutaneous screw fixation (FH and RA group), weight bearing on the injured side should be limited to "touch down" (weight of leg). Assistance with leg lifting in transfers may be necessary. Progressive weight bearing can begin according to anticipated healing. Significant weight bearing is usually possible by six weeks but use of crutches may need to be continued for three months. For patients treated by spino-pelvic fixation (ORIF group), partial weight bearing on the injured side was allowed after surgery immediately with a walking frame or crutches. Implants could be removed after consolidated fracture healing if required by the patient.

### Follow-up and data collection

The cause of injury, fracture classification, American Society of Anaesthesiologists (ASA) score for anaesthesia, number of fluoroscopy exposures, operative duration, intraoperative blood loss, surgical complications (wound infection, nerve or vessel injury) and so on were recorded.

Postoperative CT scans were used to evaluate the accuracy of screw placement in all patients. Screw perforation was classified as grade 0 (no perforation), grade 1 (perforation <2 mm), grade 2 (perforation 2–4 mm), or grade 3 (perforation >4 mm) on assessment [22].

A pelvic X-ray or CT examination was performed monthly after surgery until the fracture was completely healed. The outpatient department reviewed the images to evaluate screw positioning and pelvic fracture healing and to identify and address postoperative complications in a timely manner, such as bedsores, deep venous thrombosis of the lower limbs, and falling pneumonia.

At three, six and 12 months after injury, the Majeed score [23], which consists of seven items (pain, work, sitting, sexual intercourse, walking aids, gait unaided, walking distance) and has a maximum of 100 points, was used to evaluate functional outcomes. As some patients did not work prior to the trauma and some did not have sexual intercourse within the year prior to the trauma, the achievable maximum value varied among the included patients. To correct for this, final analysis was made using the percentage of the achievable maximum Majeed score (*e.g.,* 65 of 80 points = 81.3% in a patient who did not work before the accident).

### Statistical analysis

Statistical analysis was performed using SPSS Version 19.0 (IBM, Corporation, Armonk, NY). Student’s t test was used for continuous variables, which are expressed as the mean ± SD and minimum to maximum value, whereas the chi-square test or Fisher’s exact test was used for categorical variables. Statistical significance was defined as *p* <0.05.

## Results

### Baseline characteristics

A total of 119 patients (55 men and 64 women; age range, 15–89 years) were included in this study. A total of 92.4% (110/119) of the injuries were caused by motor vehicle collisions or falling from a height, and 65.5% (72/110) of the injuries were complicated with other injuries, such as thoracic or abdominal injuries or spine or limb fractures. A total of 7.6% (9/119) of the patients suffered from low-energy injuries; most of these patients were elderly patients with osteoporosis. The pelvic fractures of all patients were type B according to the AO classification, and type B2 was the most common, accounting for 84.0% (100/119).

Twenty-four patients chose nonoperative treatment after being fully informed: two patients were in critical condition and could not tolerate anaesthesia and surgery; one patient developed a Morel-Lavallee lesion and infection; one patient was pregnant. Forty-five patients were treated with ORIF. Among them, four patients had open fractures combined with urethral, bladder or rectal injuries. Ten patients were treated with FH. Forty patients were treated with RA; among them, eight had open fractures.

Patient characteristics are presented in Table 1. No statistically significant differences were found in the general characteristics or fracture classification between the RA group and the other three groups.

**Table 1:** Demographic data of the four groups

**Groups**	Nonoperation (*n*=24)	ORIF (*n*=45)	FH (*n*=10)	RA (*n*=40)
Sex (M/F)	8/16	22/23	4/6	21/19
Age (years)	49.5±19.5(15~89)	47.6±13.9(18~74)	53.3±16.4(29~79)	49.0±15.0(17~76)
BMI (kg/m^2^)	22.4±3.0(18.6~30.5)	22.7±2.3(18.0~27.1)	22.6±2.9 (18.2~31.6)	23.0±3.6(16.0~32.9)
Injury cause (high-/low-energy damage)	21/3	43/2	7/3^**^	39/1
Open fracture (cases)	0^*^	4	0	8
Osteoporosis (cases)	5	6	3	6
Pre-OP time (days)	/	9.7±5.4 (3~30)	8.8±4.1 (5~15)	8.9±4.9 (3~23)
Follow-up (months)	17.1±6.3 (12~34)	20.3±10.4 (12~42)	18.4±5.6 (12~30)	16.5±3.8 (12~36)
ASA grade (1/2/3/4)	9/7/4/4	20/16/9/0	8/1/1/0^*^	12/21/6/1
AO classification (B1/B2/B3)	3/18/3	3/38/4	2/7/1	1/37/2

Compared with the RA group, *p <0.05, **p <0.01

### General results of surgery

**Fig. 3:**
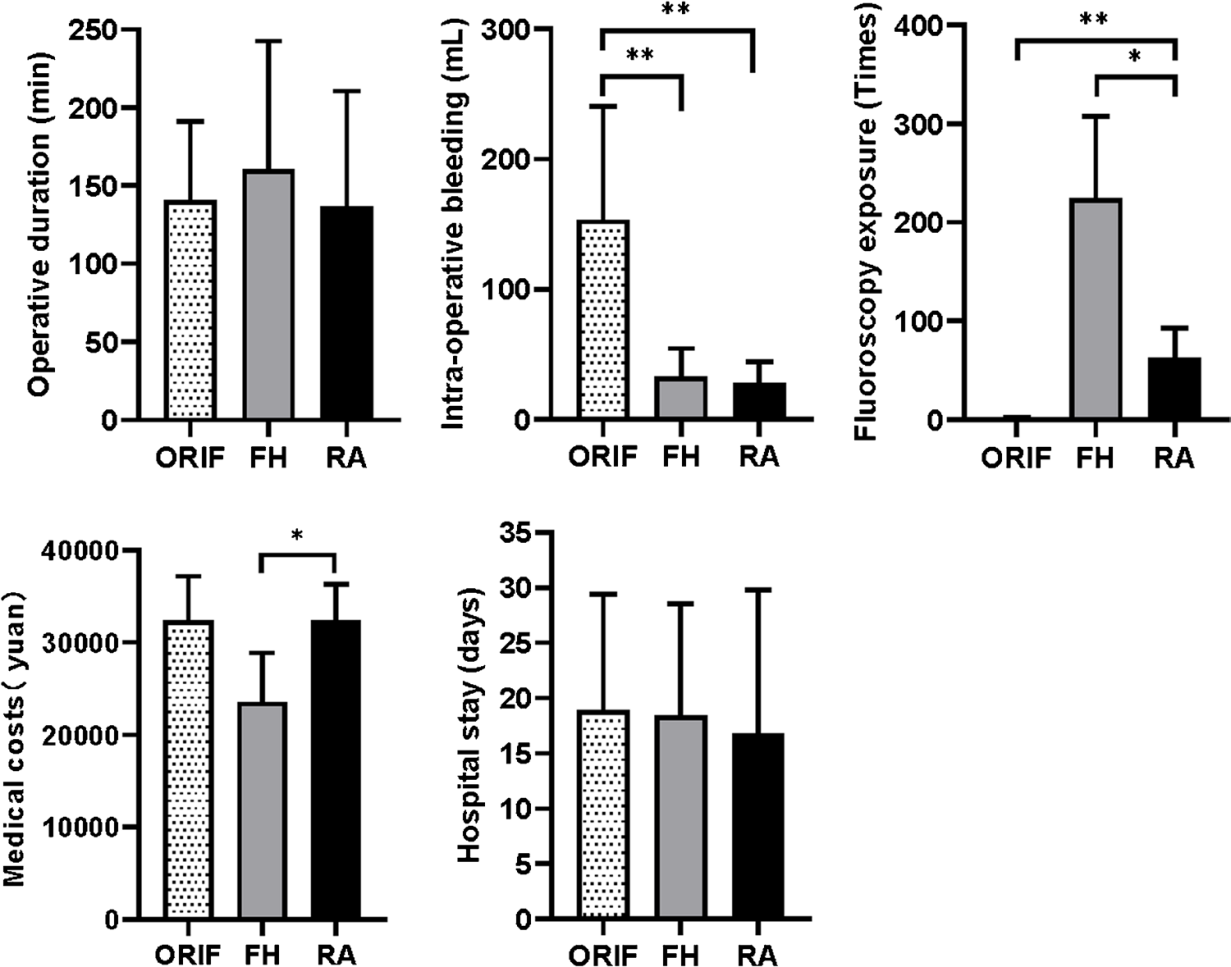
General results of surgery

As shown in Fig. 3, there was significantly less intraoperative blood loss in the RA group (27.5±16.7 mL) and FH group (32.9±21.4 mL) than in the ORIF group (153.3±87.0 mL) (both *p*<0.01). The number of fluoroscopy exposures was lower in the RA group (62.6±30.1 times) than that in the FH group (224.6±82.5 times) (*p*<0.05) but much higher than that in the ORIF group (2.2±1.4 times) (*p*<0.01). In the FH group, the accuracy of one screw was grade 1, while the accuracy of the others was grade 0. The accuracy of all screws in the RA group was grade 0. There were no cases of neurovascular injury in any of the three groups. In the ORIF group, there were five cases of wound infection (3 cases in patients treated with ilioiliac plate fixation, 1 case in a patient treated with spino-pelvic fixation, and one case in a patient with an undiscovered rectal injury before the operation), which all required debridement and antibiotics; additionally, in one case, the ilioiliac plate was removed before fracture union to control the infection. There were no cases of infection in the FH or RA group. The medical expenses were slightly higher in the RA group (32461.1 ± 3857.3 yuan) than in the FH group (23543.2 ± 5341.6 yuan) (*p*<0.05), with no significant difference from the ORIF group (32340.8±4892.0 yuan). There was no significant difference in the operative duration or length of hospital stay between the three groups.

### Clinical and functional outcomes

After more than one year of follow-up, all pelvic fractures healed. A few patients in the nonoperative group experienced fracture displacement and malunion, which led to chronic local pain and poor function. In the ORIF group, there were three cases of partial screw extraction, all of which occurred in patients with osteoporosis who had been treated with plate fixation for pubic branch reconstruction, but the patients did not feel discomfort. Patients treated with ilioiliac plate/spino-pelvic fixation mostly complained of sacrococcygeal stimulation by the internal fixation, while patients treated with pubic symphysis surgery mostly complained of pain during sexual intercourse. All patients in the FH and RA groups recovered smoothly and were able to stand, walk and take care of themselves approximately three months after the operation, and there were no symptoms of screw loosening or stimulation by internal fixation.

**Fig. 4:**
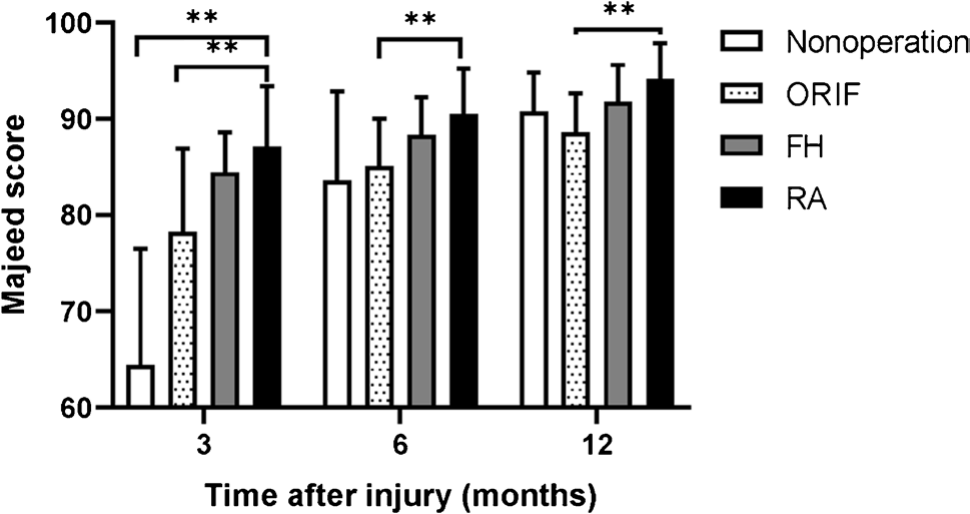
Majeed score in the four groups at 3, 6 and 12 months after injury

Three months after injury, the Majeed score was lowest in the nonoperation group (64.5±12.0), while that in the RA group (87.1±6.3) and HA group (84.4±4.2) was slightly higher than that in the ORIF group (78.2±8.7). One year after injury, the Majeed score of patients in the nonoperative group improved to 90.7±4.1, without a difference from that in the FH (91.8±3.8) and RA groups (94.15±3.7), and the Majeed score in the RA group was higher than that in the ORIF group (88.6±4.1) (Fig. 4).

The number of patients in the ORIF, FH and RA groups who finally underwent internal fixation removal was four, one and seven respectively. The four patients in the ORIF group required implant removal because of local discomfort. The removal process was successful in all patients, and there were no complications, such as infection or neurovascular injury.

## Discussion

In recent years, many scholars have put forwards the concept of “fragility fracture of the pelvis” (FFP) and conducted in-depth research on its classification and treatment [3, 24, 25]. These kinds of fractures are typically the result of a low-energy impact or may even occur spontaneously in patients with severe osteoporosis. Most FFPs are minimally displaced, but insidious progression of bone damage leads to increased displacement, nonunion and persistent instability. Percutaneous cannulated screw fixation is the best choice for the treatment of type II FFPs (nondisplaced posterior injuries with or without involvement of the anterior pelvic ring).

In this study, we found that similar low-energy pelvic fractures could also occur in young patients. In addition, if young patients with high-energy injuries also experienced fractures in other areas, such as the femur and spine, their pelvic fractures may also be nondisplaced. Notably, X-ray examination may not be suitable for diagnosing such nondisplaced pelvic fractures. Therefore, for patients with low-energy injuries but pain and discomfort in the lower abdomen or hip or patients with high-energy injuries and a poor general condition who cannot cooperate with a physical examination, pelvic CT should be performed to avoid a missed diagnosis.

When facing a nondisplaced pelvic fracture, most doctors and patients struggle with whether to treat it surgically. In this study, we found that most patients recovered within approximately four months after nonoperative treatment, but the cost was that the patients could not move freely and needed to be taken care of by others. In addition, a few patients developed fracture malunion and persistent local pain that did not gradually dissipate over time. Because many nonoperative patients were lost to follow-up or underwent follow-up by telephone without a detailed examination, the results of our follow-up observations are biased, and there might have been more complications than reported above. Consistent with other literature reports, we found that the older the patient was or the more combined injuries there were, the higher the risk of complications and the worse the effect of nonoperative treatment [2, 3]. Therefore, conservative treatment is only suitable for patients with a good physical condition and good compliance.

ORIF can allow patients to exercise early, and the incidence of surgical complications can be controlled to a very low level for experienced doctors [26]. However, there are some unavoidable problems. First, for open pelvic fractures (for example, anterior ring fractures with urethral, bladder or rectal injuries), ORIF through an anterior approach will lead to wound infection and hinder bladder or urethra repair. Second, patients often feel stimulation by internal fixation after surgery. Third, for patients with osteoporosis, the plate and screws used for internal fixation tend to loosen easily. Fourth, implant removal is challenging. Therefore, ORIF should not be used as the first choice for nondisplaced pelvic fracture patients but only be recommended for patients with displaced fractures that require open reduction.

In this study, we once again confirmed that compared with ORIF, percutaneous cannulated screw fixation causes less trauma and fewer complications. In addition, even for open fractures, the risk of infection caused by cannulated screw fixation is very low, and it does not affect operations to repair the bladder, urethra, or rectum. The patients have no obvious discomfort after the operation, and it is very simple and safe to remove the implant after fracture union. Therefore, percutaneous cannulated screw fixation has been recognized as the preferred treatment for nondisplaced pelvic fracture patients. However, free-hand empirical screw placement under fluoroscopic monitoring is challenging due to the complex pelvic anatomy and skilful correlation of fluoroscopic images and bony landmarks required. The successful development of orthopaedic robots makes the operation simple, with precise positioning, minimal invasiveness, and minimal radiation exposure. In this study, we found that the Majeed score of RA group was the highest, especially at three months after operation, which means, early outcome was better in RA group than that in Nonoperation or ORIF group. Moreover, in our analysis, the medical cost in the RA group was not higher than that in the ORIF group. Therefore, RA is the best choice for nondisplaced pelvic fracture patients.

### Surgical experiences

RA is not suitable for all nondisplaced pelvic fractures. Pelvic congenital malformation and channel stenosis would lead to surgical failure; thus, it is necessary to carefully read the three-dimensional CT reconstruction of the pelvis before the operation and design the channel position.

Although the posterior pelvic ring bears more vertical stress, its fixation is more important. However, if the anterior ring fractures are not reduced and fixed, internal pelvic rotation displacement occurs when the patient lies on one side. Therefore, for patients with anterior and posterior pelvic ring injuries, both injuries should be fixed to solve the problem of vertical and rotational instability. In clinical work, pelvic fractures with anterior ring displacement and no posterior ring displacement are more common. We chose robot-assisted sacroiliac screw fixation combined with superior ramus of pubis reduction and fixation though the Stoppa approach for those patients, which is also simple, minimally invasive and associated with few complications.

### Study limitations

This study only included cases of pelvic fractures without displacement, but displaced pelvic fractures are more common. In addition, some Majeed scores were not evaluated at the time of outpatient follow-up three, six and 12 months after the injury but were made according to the patients' memories during the later telephone follow-up; thus, there may be bias in these results.

## Data Availability

The datasets generated during or analyzed during the current study are available from the corresponding author on reasonable request.
